# Postharvest delivery of *Bacillus* G36 metabolites formulated in AgNP modifies *Salvia rosmarinus* Spenn. bioactive profiles

**DOI:** 10.1038/s41598-026-43957-z

**Published:** 2026-03-17

**Authors:** E. Fuente-González, Svitlana Plokhovska, E. Gutierrez-Albanchez, B. Ramos-Solano, F. J. Gutiérrez-Mañero

**Affiliations:** 1 Facultad de Farmacia. USP-CEU, CEU-Universities, Urbanization Montepríncipe, Boadilla del Monte, 28660 Spain; 2 Institute of Food Biotechnology and Genomics, NAS of Ukraine, Kyiv, Ukraine

**Keywords:** Silver nanoparticles, biological synthesis, postharvest elicitation, rosemary, secondary metabolism., Biochemistry, Biological techniques, Biotechnology, Microbiology, Plant sciences

## Abstract

This study explores the biological synthesis of silver nanoparticles (AgNPs) using elicitors (ML) from the beneficial bacterium *Bacillus* G36 and evaluates their potential to enhance bioactive compound production in postharvest rosemary (*Salvia rosmarinus* Spenn.). To determine optimal synthesis conditions, different ratios of ML to AgNO3 1mM were mixed, for 24 h, evaluating a range of temperatures (28–37) and pH (5, 7, 9), with and without rosemary extracts, which were characterized by TEM, FTIR, XRD and Zpotential. Two NP were selected based on size and organic crown for biological assay on rosemary. The best conditions were a 1:1 (vv) mixture of ML and silver nitrate, pH 9, 37 °C, producing nanoparticles with an average size of 7.5 nm (S3). These small AgNPs have shown great biological activity. In contrast, adding rosemary extract (RE) to nucleation media, in order to increase reductive media potential, yielded larger particles (≈ 63.9 nm) and reduced their effectiveness. Biological assays showed that S3 AgNPs significantly increased total phenol and flavonol contents in rosemary when applied in postharvest, while ML alone did not, highlighting the better effect of elicitors when formulated in NP. Remarkably, only S3 AgNPs enhanced rosmarinic acid levels by 50%. Both S3 AgNPs and live *Bacillus* G36 cells also boosted diterpene (carnosic acid equivalents) concentrations. Despite effects on bioactives, only S3 and ML treatments increased the total antioxidant capacity of the extracts. Overall, the study demonstrates that biosynthesized S3 AgNPs from *Bacillus* G36 metabolites offer a sustainable and efficient approach to producing small, bioactive nanoparticles capable of improving and maintaining valuable phytochemicals in rosemary postharvest.

**Supplementary Information:**

The online version contains supplementary material available at 10.1038/s41598-026-43957-z.

## Introduction

*Salvia rosmarinus* Spenn., also known as *Rosmarinus officinalis* L. or its common name, rosemary, is a herbaceous plant widely used from ancient times in the treatment of numerous conditions and diseases, and recently studied for its antioxidant and anti-inflammatory effects, also useful in food preservation^1^. Reported effects of rosemary extracts and their isolated bioactive compounds include the regulation of reactive oxygen species (ROS) levels, stimulation of Nrf2-mediated antioxidant enzyme synthesis^2,3^, and modulation of inflammatory responses through inhibition of COX-1 and COX-2 activity, as well as regulation of cytokine production^2–4^.

Rosemary is also known for beneficial effects on diseases such as depression, Alzheimer’s disease, metabolic syndrome and diabetes, rheumatoid arthritis, neuropathic pain, or cancer^5–7^. These effects are attributed to its active compounds of terpene and phenolic nature, all of which are secondary metabolites^8^. Therefore, improving the production of active metabolites in medicinal plants such as rosemary is of interest to the pharmaceutical industry.

Plant’s secondary metabolism is an adaptive metabolism to overcome adverse situations the plant may face along its life cycle^9^, therefore, it’s inducible. Thus, secondary metabolite concentration fluctuates according to environmental conditions, but at the same time, with the proper stimuli, molecule levels can be stabilized^10^. Hence, finding active agents to trigger plant metabolism is a challenge for the pharmaceutical industry as these secondary metabolites hold an enormous market potential^11^. One of the traditional approaches for this purpose includes certain bacterial strains beneficial for plants, known as Plant Growth-Promoting Bacteria (PGPB); among these *Pseudomonas fluorescens* N21.4, *Bacillus sp*. H47 or *Bacillus amyloliquefaciens*, have demonstrated their capacity to trigger plant metabolism, enhancing adaptive responses in various plant species, including *Arabidopsis thaliana*, tomato, and others with valuable bioactive compounds such as soybean, blackberry, strawberry, olives or raspberry^12–17^.

PGPB are able to alter plant’s hormonal balance, enhance immune and defense system and trigger secondary metabolism, as a result of organic molecules produced by bacteria known as elicitors, which penetrate the plant, interacting with specific receptors and triggering the systemic response^12,18,19^. Among the most recent approaches to this goal, nanotechnology appears as an interesting alternative^20^. Nanoparticles (NP) have generated great interest in recent years due to their unique physicochemical properties and wide range of applications. Due to their nanometric size (1–100 nm), they have a large surface to volume ratio and exhibit enhanced catalytic, magnetic, electrical, mechanical, optical, chemical, and biological activities compared to larger particles^21,22^.

Nanoparticle synthesis can be physical, chemical, or biological, always orientated to chemical compound reduction. However, physical and chemical methods are hazardous and very contaminating, causing great environmental problems therefore, biological synthesis presents a better option for nanoparticle formulation, and can be developed by using plant, bacteria, fungi, yeast and algae natural reducing compounds^23,24^.

In biological synthesis, the reducing agent converts Ag⁺ to Ag⁰, and this agent itself, along with other production parameters such as medium pH, temperature, pressure, metal salt concentration, and contact time, will determine the shape, size, and physicochemical properties of the nanoparticles, as well as the efficiency and yield of nanoparticle formation^25,26^. The organic reducing matter remains on the nanoparticle surface and plays an important role in functionalizing the nanoparticle, thereby determining its properties and reactivity^27^. Biological synthesis can be carried out either using plant extracts, which possess strong reducing power due to their bioactive molecules, or through microbiological agents employing extracellular or intracellular components from fungi or bacteria^27–29^.

Despite the high reducing power of plant extracts, no biological activity on the plant is specific to the molecules contained in the extract in general. Interestingly, some bacterial elicitor molecules able to trigger plant metabolism are capable of reducing metal ions, and thus, can mediate nanoparticle synthesis and remain on the organic crown to trigger plant metabolism^27–31^. This approach enhances the delivery efficiency of the elicitors and their penetration into plant tissues via cell walls, stomata, and plasmodesmata, thanks to the nanoscale size^34^. Thus, this technique could provide unique AgNPs depending on the reducing agent used. Therefore, the selection of an appropriate reducing agent may lead to specific biological effects, as nanoparticles can behave as carriers for elicitors. Nevertheless, the higher antioxidant potential of plant molecules represents a solid material for green synthesis^32^.

Previous studies with other beneficial *Pseudomonas* strains have demonstrated that bacterial metabolites can synthesize AgNPs^33^ and exert biological activity in plants. Furthermore, when applied in postharvest treatments, these nanoparticles have been shown to stimulate secondary metabolism in rosemary, enhancing its antioxidant and anti-inflammatory potential^34^. Performing the treatment on postharvest material not only improves reproducibility for industrial applications but also facilitates extension of the method to other plant species.

Therefore, in this study, we hypothesized that bacterial metabolites (elicitors) from *Bacillus* G36 are capable to reduce Ag^+^ to Ag^0^ and synthesize nanoparticles with biological activity that could enhance secondary metabolism in rosemary increasing bioactive contents when applied during the postharvest stage. Secondly, with the aim to use rosemary extract’s reducing potential, we explored AgNO3 reduction with a combination of both elements to improve efficiency of the process. To this end, we first determined the biological synthesis conditions of AgNPs using elicitors from the beneficial strain *Bacillus* G36 and rosemary extract aiming to cause a synergistic increase in reductive potential, as rosemary is rich in antioxidant and reducing components, phenolic compounds and flavonoids. After nanoparticle characterization, biological activity was assessed in rosemary evaluating the enhancement in bioactive compounds when delivered to detached rosemary branches (Table 1).

**Table 1:** FTIR analysis of the functional group of the samples studied

Wavenumber (cm⁻¹)	Intensity	Vibrational assignment
3912–3660	Broad, sharp peak	O–H
3400–3200	Moderate-strong, well-defined peak	N–H/O–H
2920–2840	Moderate, well-defined peak	Alkyl C–H
~ 1740	Strong, sharp peak	Ester/Aldehyde C = O
1650–1630	Intense, medium-narrow, may overlap peak	Amide/Acid C = O
1550–1510	Moderate-strong, medium-narrow peak	N–H
1450–1375	Moderate, medium, not sharp peak	C–H
1260–1000	Moderate-strong, multiple peaks	C–O
900–700	Weak-moderate, sharp peak	C–H aromatic
~ 520	Weak, poorly defined peak	C–X halogen

## Results

### Silver nanoparticles synthesis and characterization

Determination of the best conditions for NP synthesis was set after experiment 1 (shown in table 1SM). The best nucleation temperature was 37 °C, and the best pH was 9.

Bacterial metabolites (ML) at pH 5 lead to no color change in media, indicating that nanoparticle synthesis did not take place; similarly, nucleation at 28 °C was not suitable for NP synthesis in any of the tested conditions. However, when ML was adjusted to pH 7 or 9, and nucleation took place at 37 °C, AgNP were effectively synthetized for ratios S1, S4 (Figure SM.1) and S3.

The best condition for AgNP synthesis was achieved by mixing the same volume of AgNO_3_ and bacterial metabolites at pH 9 (S3). This was determined considering darkness of reaction to bare eye, and UV absorption at 420 nm (Figure SM.2).

The combination of ML and RE for AgNP synthesis in experiment 2 (Table 2) showed interesting results. Nanoparticles’ presence was confirmed in every sample by UV-visible absorption spectrum with maximum peak absorption between 420 and 450 nm; peaks were more intense with higher RE concentration. Samples from ratios S3R01, S3R05, S3R1 and S3R3 needed to be diluted 1/5 to observe the peak at maximum absorption (Figure SM.3).

**Table 2:** Ratios used for silver nanoparticles synthesis in experiment one, using 1 mM silver nitrate and ML at different pH levels. Every nucleation condition was tested at 28 and 37 °C; 120 rpm during 24 h and light conditions.

Ratio	AgNO_3_ 1mM (mL)	ML (mL)	pH	ºC
S1	1	5	pH 5	28 °C 37 °C
S3	3	3		
S4	4	2		
S1	1	5	pH 7	
S3	3	3		
S4	4	2		
S1	1	5	pH 9	
S3	3	3		
S4	4	2		

**Fig. 1:**
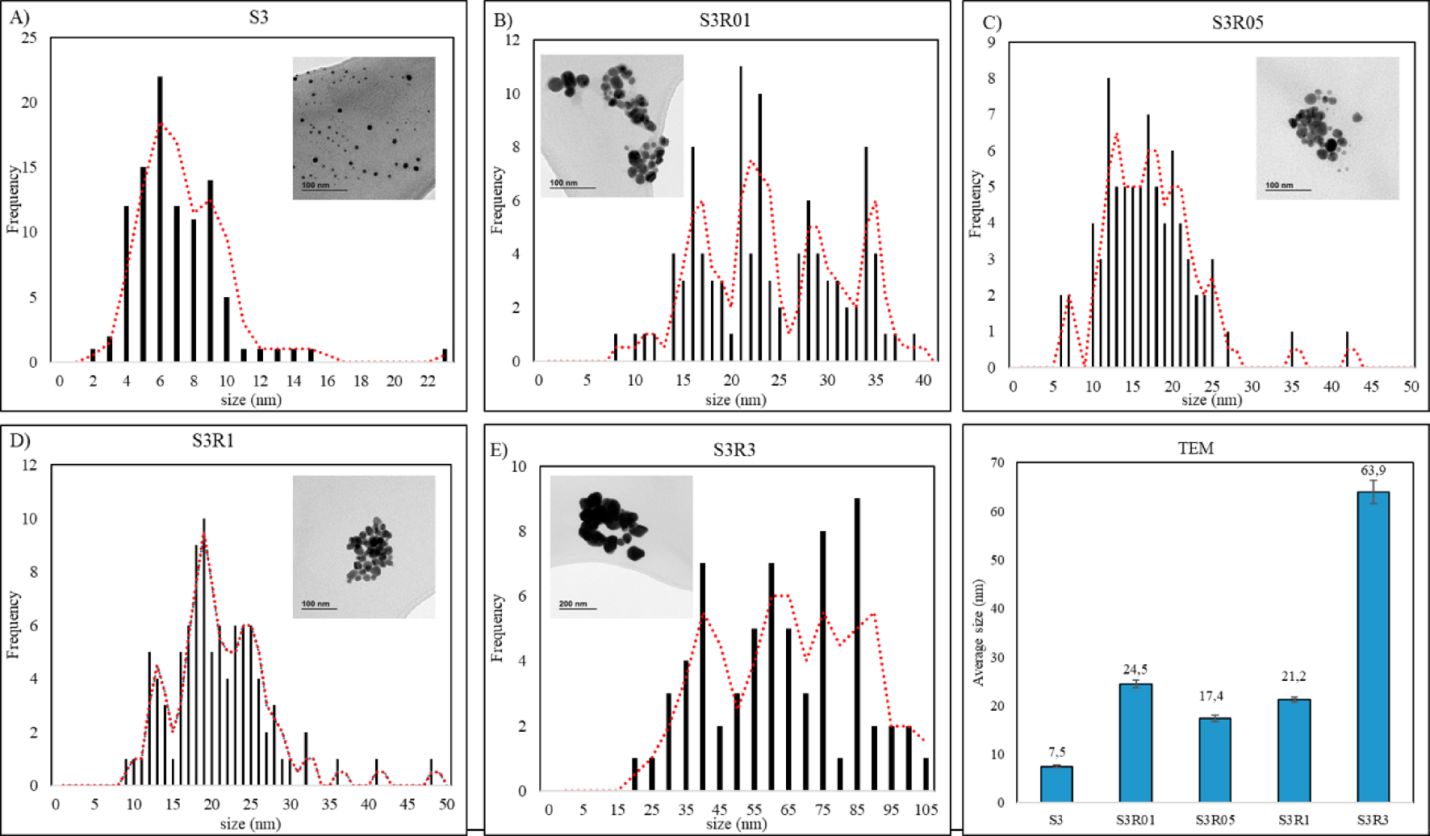
(**A**–**E**) Characteristics of AgNPs in TEM images and histograms of the particle size distribution of AgNPs. Bar: 100 nm, except for S3R3, that was 200 nm TEM. F) Average particle size represented with mean ± SD, from 100 NP.

TEM analyses showed spherical nanoparticles in samples S3, S3R01 and S3R05, but this spherical morphology disappeared in ratios S3R1 and S3R3, consistent with higher RE on the sample (Fig. 1). The smallest NP were obtained with bacterial metabolites only (S3), showing an average diameter of 7.5 nm; the largest were obtained with RE only (S3R3) with an average diameter of 63.9 nm (Fig. 1).

**Fig. 2:**
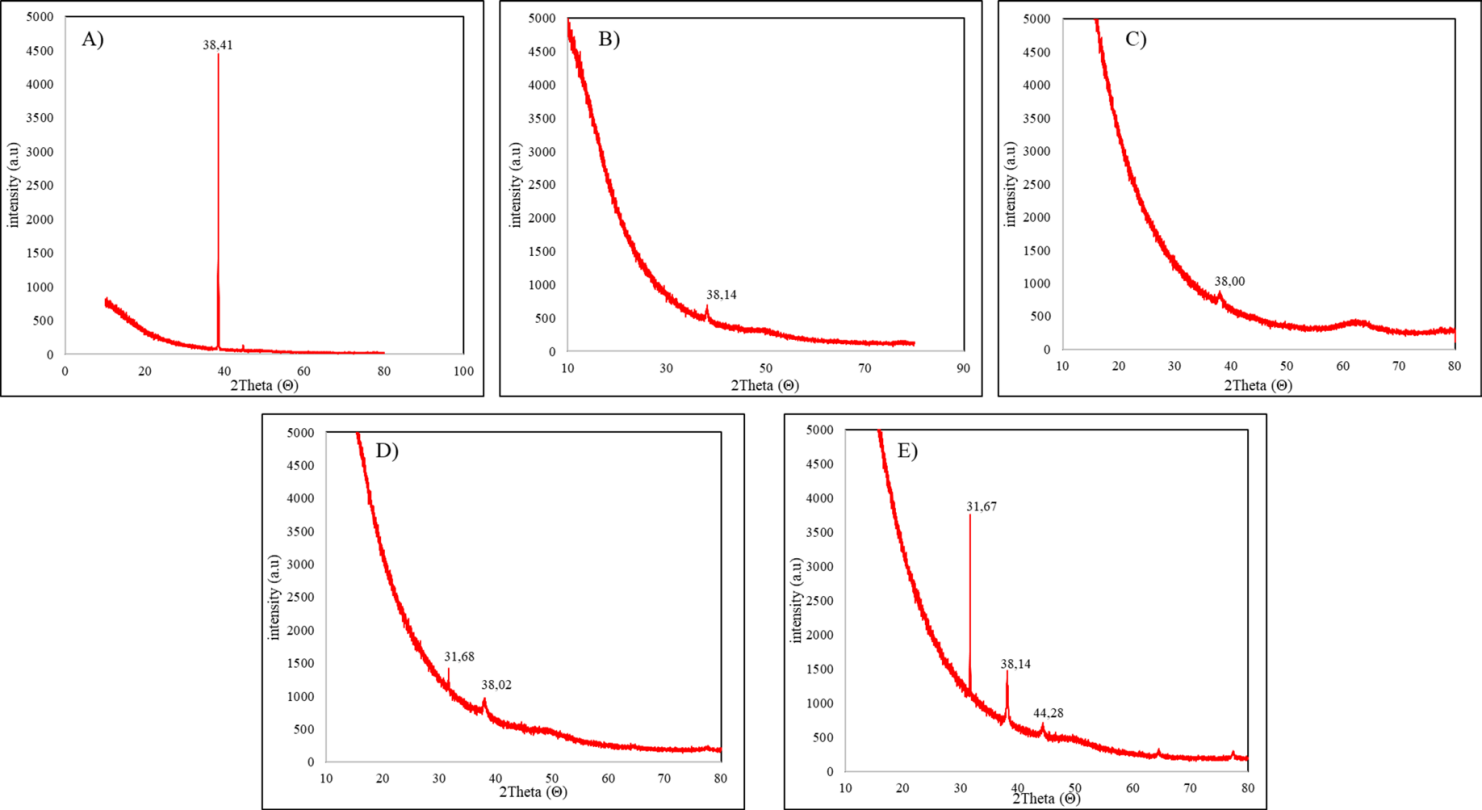
Characteristics of synthesized AgNPs in XRD analysis (**A**) S3 (**B**) S3R01 (**C**) S3R05 (**D**) S3R1 (**E**) S3R3.

The crystalline structure of the bio-synthesized AgNPs was determined using XRD analysis (Fig. 2). S3 presented just one peak at 38◦, which represents just Ag^0^. All nanoparticles synthesized with rosemary extract presented similar results for XRD, with lower intensity peaks than S3. Characteristic Bragg reflection peaks at 2θ values were observed at 31.6◦, 38.0◦, 38.1◦, 38.4◦ and 44.2◦, a profile associated to a face-centered cubic structure of AgCl crystals (Ref. no. 31-1238). (Fig. 2).

**Fig. 3:**
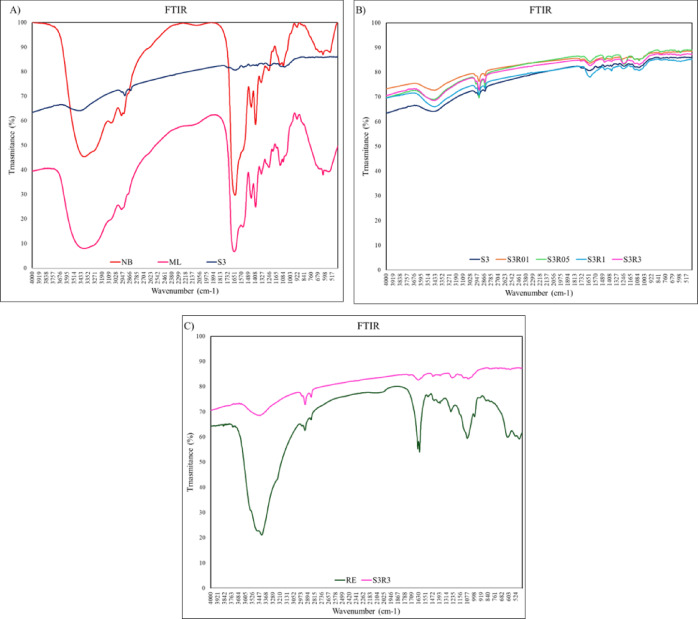
(**A**) Characterization of components on the surface of S3 AgNP, broth (NB), ML and RE. The nutrient broth and bacterial filtrate before synthesis are shown in pink and red, respectively; AgNP S3 spectra after synthesis in dark blue; (B) Components characterization of the surface of all AgNP of experiment 2; (**C**) RE (dark green) organic matter characterization before synthesis and AgNP S3R3 in light pink.

FTIR profiles are shown on Fig. 3. The spectra of the raw bacterial metabolites (ML) before NP synthesis, raw culture broth without bacterial growth (NB) and S3 NP (Fig. 3A) were different, indicative of different chemical composition; ML showed lower transmittance than NB, indicative of exclusive bacterial metabolites released to culture media; S3 profile was different to ML indicating that only a fraction of metabolites have been incorporated along the nucleation process to the NP surface. RE (Fig. 3C) was different to ML profile; also, among all organic matter present on the RE, only specific molecules are incorporated to the NP. Finally, when all NP profiles from experiment 2, with and without RE, are compared (Fig. 3B), all show a similar profile, being the S3 without RE the one that determines the profile of all, and also the one that incorporates more organic matter based on lower transmittance.

**Fig. 4:**
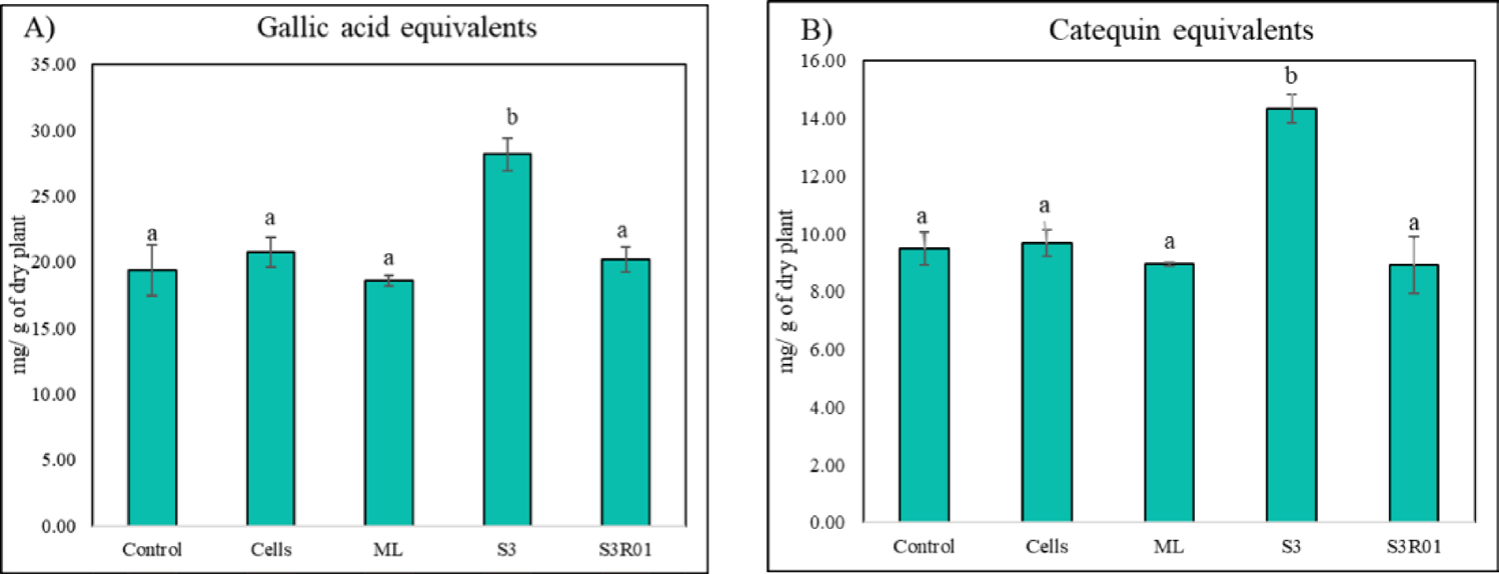
(**A**) Total phenols (gallic acid equivalents) and (**B**) total flavonols (catequin equivalents) in rosemary extracts under the described treatments. Values correspond to the mean ± SD (*n* = 3). Different letters indicate statistically significant differences among treatments as determined by one-way ANOVA followed by Tukey’s HSD post hoc test (*p* < 0.05).

The different functional organic groups are identified in Table 3 following FTIR Functional Group Database Table with Search https://instanano.com/all/characterization/ftir/ftir-functional-group-search/)

**Table 3:** Ratios used for silver nanoparticles synthesis in second experiment, using 1 mM AgNO_3,_ ML at pH 9 and 37 °C nucleation temperature. Nucleation conditions were developed during 24 h, continuous light conditions and 120 rpm agitation.

Ratio	AgNO_3_ 1mM (mL)	ML (mL)	RE (mL)
S3	3.0	3.0	-
S3R01	3.0	2.9	0.1
S3R05	3.0	2.5	0.5
S3R1	3.0	2.0	1.0
S3R3	3.0	-	3.0

Zeta potential measurements revealed negative surface charges for S3 and S3R01 nanoparticles, -26,22mV and-18,23mV respectively. Lower values indicate higher stability and better nanoparticle dispersion.

## Biological assay results

Only S3 Ag-NP significantly increased total phenols (Fig. 4A) and total flavonols (Fig. 4B) while cells, raw metabolites (ML) and S3R01 NP did not. No significant differences were observed between the control and the negative control with AgNO₃ 1mM in similar amounts to S3 AgNP; however, slight significant decreases were found in flavonols content (Figure SM. 4).

**Fig. 5:**
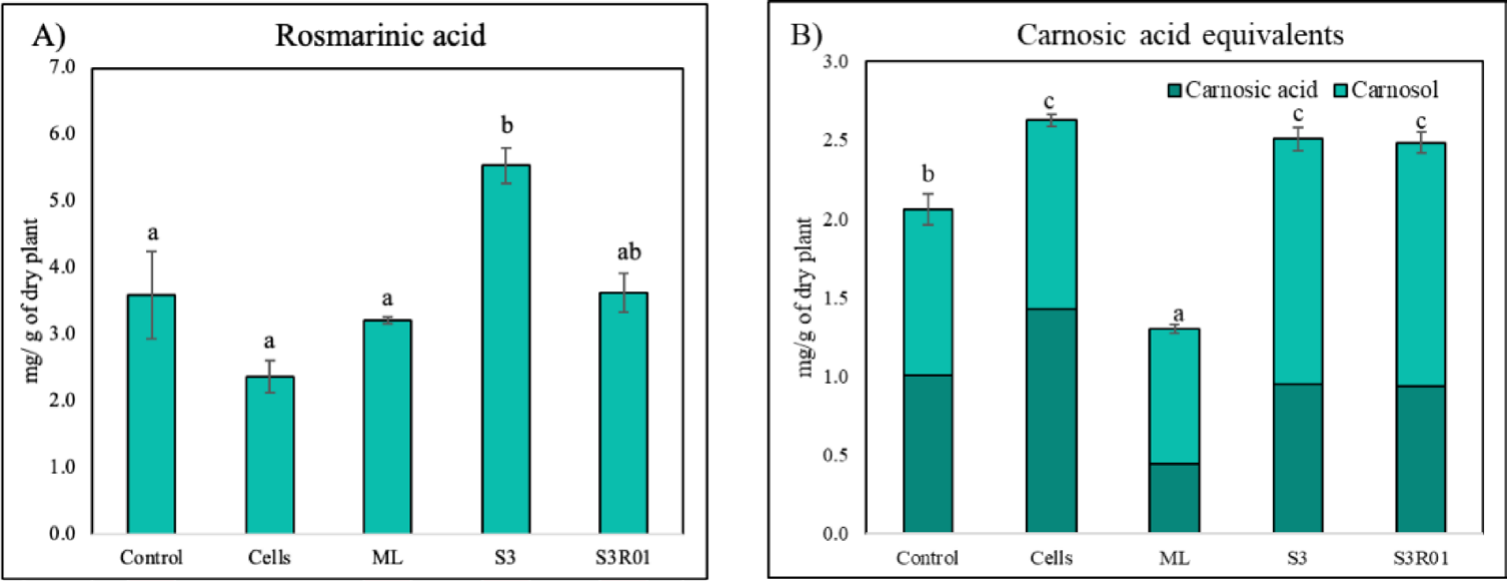
(**A**) Rosmarinic acid (mg of rosmarinic acid per gram of dry plant). And (**B**) carnosic acid equivalents, (carnosol and carnosic acid), (mg per gram of dry plant). Values correspond to the mean ± SD (*n* = 3). Different letters indicate statistically significant differences among treatments as determined by one-way ANOVA followed by Tukey’s HSD post hoc test (*p* < 0.05).

Data from HPLC analyses revealed that S3 AgNP significantly increased rosmarinic acid contents by a rough 50% as compared to control or any other treatment. Regarding the diterpenes group, a higher concentration of carnosic acid equivalents (carnosic acid plus carnosol) was found in extracts from Cells, S3 and S3R01 treatments, reaching similar values, but significantly lower concentration in the ML treatment (Fig. 5).

**Fig. 6:**
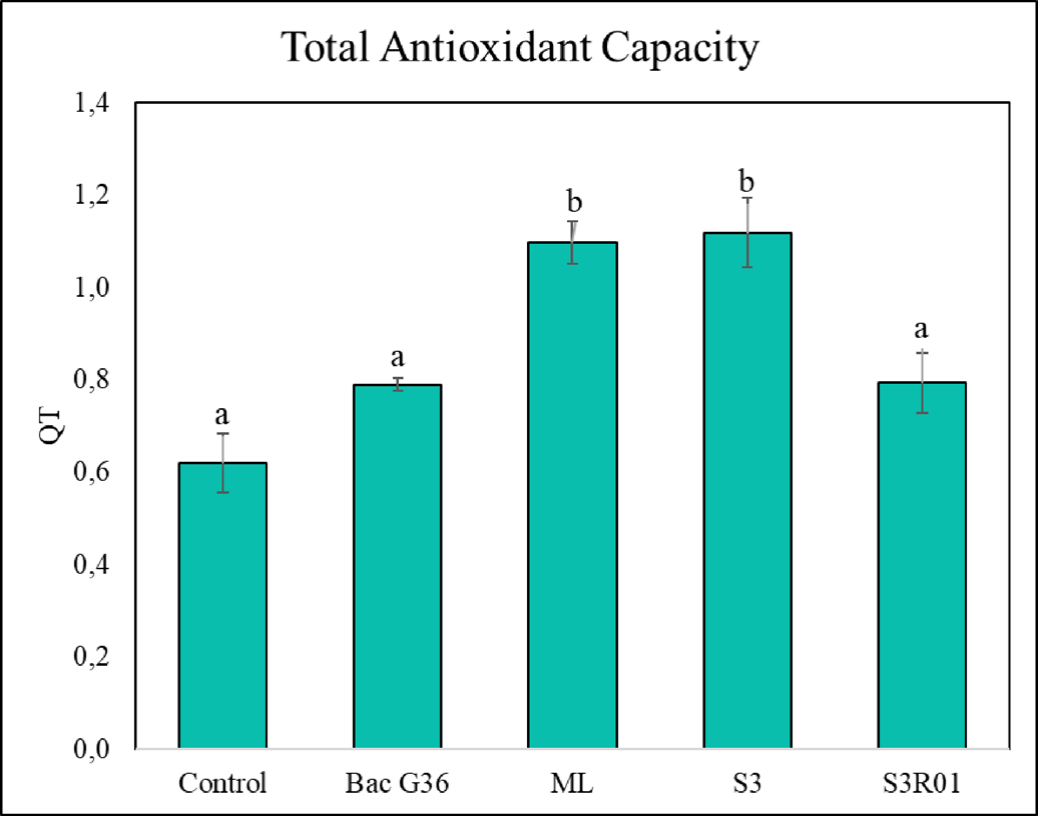
Total antioxidant capacity (TAC) was expressed as QT (total electric charge transferred during the oxidation reaction) in µC. Values correspond to the mean ± SD (*n* = 3). Different letters indicate statistically significant differences among treatments as determined by one-way ANOVA followed by Tukey’s HSD post hoc test (*p* < 0.05).

Antioxidant capacity was significantly increased in S3 and ML rosemary extracts as compared to the control (Fig. 6).

## Discussion

The use of nanotechnology to enhance secondary metabolites with pharmacological activity, relevant for the pharmaceutical industry, is reinforced and supported by the results obtained in the present study. The working hypothesis is confirmed by the successful reduction of Ag⁺ by *Bacillus* G36 metabolites, resulting in the synthesis of AgNP with a unique organic crown endowed with biological activity, as demonstrated by the increase of bioactive molecules in rosemary. This is an exclusive effect of the S3 nanoparticle, since neither the bacterial strain nor the bacterial metabolites alone are capable of enhancing phenols or diterpenes in *Salvia rosmarinus* Spenn, thereby conferring greater potential to the phytotherapeutic product. Furthermore, the effectiveness of postharvest applications constitutes an efficient method for industry, as it can be applied to plants of different origins and agronomic management conditions^34^.

The conditions established for NP synthesis determine their size, shape, and organic cover^27,35^. Among the multiple conditions tested, pH and temperature proved to be key for NP synthesis, with neutral and basic pH of the ML being more suitable for active Ag⁺ reduction than acidic pH, conversely to other reports showing that lower pH leads to smaller size and nanoparticle agglomeration. These findings confirm that the ionization state and temperature of the molecules strongly condition the reductive potential of organic molecules in the media and that the most suitable pH may vary depending on the nucleation medium and the reductive molecules present^36,37^. In addition, the ratio of bacterial metabolites to AgNO₃ markedly determined NP synthesis^33^ making it crucial to establish the optimal ratio to achieve the best synthesis conditions^37^. Consequently, and after testing several pH value, ratios and temperatures, the best conditions for NP synthesis employing *Bacillus* G36 metabolites were established for equal volumes of metabolites to AgNO_3_ (S3), pH 9, and 37 °C nucleation temperature.

In order to further improve the reduction potential of bacterial metabolites under the conditions determined in Experiment 1, Ag bioreduction was addressed under a novel hypothesis, seeking a synergistic potential between RE and bacterial metabolites. Green synthesis has proven effective for Ag⁺ reduction^21,30,38^. Similarly to the ratio of bacterial metabolites to NO_3_Ag, plant extract (RE) concentration determines the duration of the nucleation process, which progresses more rapidly as the amount of green extract increases. This effect is attributed to the accelerated bioreduction and stabilization of nanoparticles, driven by the higher abundance of reducing molecules present in the extract^32,37^. Experiment 2 supports this notion, as RE nanoparticles (S3R01, S3R05, S3R1 and S3R3) showed a markedly faster color change than nanoparticles without RE (S3), consistent with the higher reduction potential of the medium.

Since the ratios of bioreductants to Ag^+^ affect the nucleation process^37,39^, three parts of Ag were fixed, and several combinations of ML with RE were evaluated, prioritizing ML presence. Results indicated that RE changed the physicochemical properties of AgNPs, leading to an increase in absorption peaks between 420 and 450 nm, concomitant to higher number of nanoparticles. TEM analysis showed that RE led to larger NP (63.9 nm) compared to the slower synthesis mediated by bacterial metabolites, which produced NP around ten times smaller (7.5 nm), with greater diffusion and penetration capacity and a higher surface-to-volume ratio^30,40^. While there are contradictory reports regarding the effect of plant extracts on nanoparticle synthesis, some suggesting a faster nucleation yielding smaller and more stable NPs^41^, others indicating that excess organic matter can hinder nucleation and produce larger aggregates^42^, our results clearly support the latter scenario. These findings highlight that the type and composition of plant extracts critically influence the size and properties of the resulting nanoparticles, underlining the need to optimize synthesis conditions according to the specific extract and the intended application.

Irrespective of NP surface, the combination of RE with ML produced NP with similar organic crowns, revealing that all NP are endowed with similar molecules on their surface, ML-RE combination resulted in a disadvantage in terms of nanoparticle characteristics (size and shape and crown). Since the FTIR profile was similar for all nanoparticles, it can be concluded that the organic groups fixed to the nanoparticle crown were comparable among samples; however, in S3, the organic matter likely derived exclusively from *Bacillus* G36. RE incorporation to nucleation media had resulted in a decrease in organic matter fixation although this reduction was not proportional to the RE amount, suggesting that RE could saturate the fixation sites on the nanoparticle crown. This saturation may lead to larger, less stable, and more aggregated nanoparticles, consequently reducing their efficiency in penetrating and activating secondary metabolism. This is because, besides their lower penetrating capacity, they exhibit lower proportion of bacterial-derived organic matter, which is the active component on the plant^37,43^.

Since the combination of ML with RE produces nanoparticles coated with organic matter from both sources, S3 and S3R01 nanoparticles were selected to study the enhancement of secondary metabolite production in postharvest rosemary, justified by the fact that the organic matter coating the nanoparticle is mainly derived from bacterial elicitors^44^ despite the larger size of S3R01.

The results of the biological assay demonstrate that S3 and S3R01 activity markedly differ, despite their similar organic matter profile (FTIR), leading to the conclusion that the size of S3NP is determinant for the biological effect, as S3NP increase total phenols and flavonols, while S3R01NP do not^26,35^. Exclusive increases in rosmarinic acid were found in rosemary treated with S3-AgNP, further supporting effects based not only on bacterial metabolites but also on NP size. Rosmarinic acid was significantly enhanced only by S3-AgNP, but neither by cells nor by ML, confirming that NP size allows greater penetration^43,45^ which is not achieved by the non-formulated metabolites present in ML or produced by the cells. Rosmarinic acid enhancement has also been reported in NP obtained with a similar synthesis process using *Pseudomonas shirazensis* metabolites as reducing agents^34^. In line with this, S3 nanoparticles enhanced the antioxidant potential of rosemary extract similarly to ML, whereas S3R01 nanoparticles did not exert such effect. This again confirms that the specific formulation of S3 may favor interactions that preserve or potentiate the redox-active compounds present in the extract, enhancing its health potential^34^.

On the other hand, both nanoparticles induced an increase in diterpenes (carnosic acid equivalents), which indicates that although S3R01 nanoparticles do not activate the phenolic synthesis pathway, they are able to stimulate other secondary metabolism pathways. This confirms that bacterial elicitors capable of activating diterpene synthesis are present in the organic crown of both NP. It is also remarkable that ML alone significantly reduced carnosic acid equivalents, even below control plant levels. This suggests the existence of other bacterial active molecules, not retained in the NP crown, that are able to activate metabolic pathways downstream of diterpenes, probably increasing other metabolites such as triterpenes^46,47^. Interestingly, both nanoparticles and bacterial cells increased diterpenes, suggesting that the terpene pathway is more sensitive to bacterial stimuli, as it responds to bacterial cells and, that the molecules involved in this stimulation are present in the NP organic crown.

On the other hand, the increase in diterpenes is unique to the organic crown of G36-AgNP and opposite to the reported effects of AgNP from *Pseudomonas* NFV3, which reduced carnosic acid concentration in rosemary extracts while increasing triterpenes of the asiatic acid group, thereby enhancing the in vitro anti-inflammatory potential of treated extracts^46^. These results confirm the differences in biological activity between the two types of nanoparticles synthesized under different conditions and emphasize the importance of size and of the organic matter that coats and functionalizes the nanoparticle^37^.

Overall, results confirm the hypothesis, showing that *Bacillus* G36 metabolites can reduce silver during silver nanoparticles synthesis, and this AgNPs applied in postharvest rosemary induce and increase in its secondary metabolites greater than cells or ML. Furthermore, no significant limitations are anticipated for postharvest delivery, as AgNP application to harvested branches and extraction of active compounds are all straightforward, safe, and with low environmental impact; moreover, environmental conditions do not affect the procedure, since treatments are to be applied to postharvest plants under controlled conditions in a closed system. Potential hazard due to Ag oxidation is low based on the Z-potential value, indicative of NP stability.

However, RE resulted in lower nanoparticles functionalization and biological activity, modifying size and crown unfavorably. In summary, the novelty of our results rely on (i) the uniqueness of the bacterial strain that provide active biomolecules (elicitors), (ii) formulation of these elicitors in AgNP, and (iii) delivering in postharvest period will ensure higher and consistent yields from materials from different origins, (iv) delivering in the postharvest period extends its applicability to other species with pharmacological or industrial interest. Therefore, the S3 Bio-AgNP constitutes a useful and sustainable tool to increase and stabilize bioactive concentrations in rosemary when applied during the postharvest period.

## Materials and methods

### Bacterial strain and treatments

*Bacillus sp.* G36 (G36 from this point onward) a Gram positive strain isolated from the rhizosphere of *Pinus pinea* from Sierra de Aracena, Sevilla^48^ was used.

This strain was preserved at -60 °C in 20% glycerol. For starting the assay, it was transferred to PCA plates and incubated at 28 °C for 24 h. Then, bacterial cells were grown in Nutrient Broth for 24 h, at 28 °C under shaking at 120 rpm, to prepare a pre-inoculum by adjusting at OD = 1 (600 nm). Then, metabolites for NP synthesis were obtained from a 24 h culture starting with a preinoculum at 1% in the same conditions as above^39^.

After 24 h, a standardized culture was obtained. Bacterial cells were separated from metabolic liquid (ML) by 20 min centrifugation at 5000 rpm, 4 °C, using a centrifuge LISA AFI-C200R-E and then filtered by 0.2 μm cellulose filtres to ensure absence of bacterial cells. Pellet of bacterial cells (Cells) resuspended in the same volume of ultrapure water (Milli-Q, Millipore). ML was further used to synthesize AgNP as described in^39^.

### Rosemary plants and rosemary extract

Three-year-old *Salvia rosmarinus* Spenn. plants used in this study were provided by “El Ejidillo Viveros Integrales S.L. Segovia, Spain. (https://ejidillo.com/*)”.* Rosemary plants were planted in experimental plots of University San Pablo CEU (Boadilla del Monte, Madrid, Spain) in 2023, where they grew under natural photoperiod and natural water regime.

Rosemary extract (RE) preparation was developed as in^49^ with modifications: rosemary stems were collected and dried at 40 °C during 72 h; 0.5 g gram of powdered dry leaves were soaked in 10 ml of 75% ethanol and incubated overnight at room temperature and darkness. After 1100 rpm 10 min centrifugation to separate solid debris, extract was filtered with 0.2 μm acetate cellulose filters and brought to dryness on a Buchi rotavapor R-300 BUCHI (11R300211V121). Dry extract was weighted and resuspended in 100% ethanol, using the same volume that was recovered after centrifugation.

### Biological synthesis of AgNPs

A two-stage procedure for AgNP synthesis was designed. First, using bacterial metabolites (ML) as bioreductants to determine the best conditions^39^ for NP synthesis, and secondly, adding rosemary extracts (RE) to further improve bioreduction of Ag^+^.

Hence, two different assays were carried out to determine the best conditions for AgNPs synthesis:

1. Firstly, G36 elicitors (ML) and AgNO_3_ 1mM in different ratios, pH and temperature conditions were tested as described in Table 2. The best conditions were selected based on NP size (smallest average diameter) and frequency of size distribution.
2. Secondly, once the best conditions were selected from the first experiment, a new experiment was carried out modifying the composition of bioreductans using bacterial metabolites and Rosemary Extract (RE).

Experiment 1. ML was adjusted to pH 5, 7 and 9. Then ML were then mixed with different volumes of AgNO_3_ 1 mM as indicated in Table 1; each condition is indicated by S (supernatant = ML) followed by a number indicating the volumes of AgNO_3_ used (i.e. S1 indicates 5 volumes of ML and 1 volume of AgNO_3_). To determine the best nucleation temperature, two identical batches were prepared, and they were incubated for 24 h at 28 °C and 37 °C respectively, and 120 rpm shaking, under continuous light.

Experiment 2. In the second experiment, S3, pH9, 37 °C nucleation temperature was used as base to introduce rosemary extract (RE) as modulator of reducing potential; The 3 volumes of AgNO_3_ 1mM were fixed, introducing variations in the 3 volumes of reducing agents. The 3 mL of reducing agents are shown in Table 2. These conditions are named by S (supernatant), 3 (3 volumes of ML), R (for rosemary extract), and a number indicating mL of RE (Table 2).

After synthesis was finished, NPs were washed and lyophilized. The dry residue was weighed, and a working solution of 60 ppm was prepared^34,39^.

### Silver nanoparticles characterization

Nanoparticles presence was first confirmed by changing color media from light yellow to dark brown indicative of Ag reduction and then confirmed by carrying out UV-visible absorption spectrum between 200 and 800 nm at 1 nm resolution with SPECTROstar Nano spectrometer (BMG LABTECH, Germany). Presence of reduced Ag^0^ was confirmed because of an absorption increase between 420 and 450 nm.

Size distribution and morphology of AgNP was estimated by TEM analysis using Thermo Fisher Scientific Prisma E y xT Microscpe Control V16.2.2 software. The measurements were carried out by adding a drop of nanoparticles suspension on aluminum and magnesium mount, letting them dry before microscope observation. After taking some pictures, Everhart-Thornley Detector (ETD) for secondary electrons at 30 kV and a spot size of 2.0 (= 24 pA) obtain high-resolution images. TEM analyses were carried out at ICTS-CNME (https://cnme.es/). One hundred nanoparticles in each sample were measured to calculate the size distribution.

The organic crown of AgNP surface and therefore, the functional groups responsible of AgNP stabilization were detected by Fourier Transform Infrared spectroscopy (FTIR) in HBR pellets. The samples were scanned using a Spectrum Two FTIR Spectrometer (Perkin Elmer) with a resolution of 4 cm⁻¹ and a range of 450–4000 cm⁻¹. The analyses were carried out at SIDI (https://www.uam.es/uam/en/sidi/unidades-de-analisis/unidad-analisis-estructural-molecular/ftir*).* In addition to the synthesized nanoparticles, FTIR was also used to study the functional groups present in the nutrient broth used for bacterial growth (CN), in the supernatant obtained after filtration (ML), and in the rosemary extract used to enhance the reducing capacity of the medium (RE).

Lyophilized AgNP were also studied in XRD using Brunker D8 and a LynxEye detector, working at a voltage of 40 kV and a current of 40 mA, with a scanning speed of 0.01 s⁻¹ (https://www.uspceu.com/investigacion/servicios-apoyo/servicio/difracción-de-rayos-x*).*

### Zeta potential

Zeta potential was determined by ELS using PALS technology in a BeNano 90 Zeta analyzer under aqueous conditions and analyzed using the Smoluchowski model.

### Biological assay on rosemary

Based on obtained results after AgNP characterization, two nanoparticles were selected for biological assay on postharvest rosemary leaves.

Apical stems (15 cm long) from 3-year-old rosemary plants were harvested randomly from the 12 plants in 3 plots; three stems from different plants were pooled constituting a replicate, and 3 replicates per treatment were used. Right after harvesting, stems were sprayed with a volume of 5 mL of each treatment: G36 Cells, G36 ML, AgNP S3 at 60 ppm, AgNP S3R01 at 60 ppm; an equivalent volume of AgNO_3_ 1mM was also tested as negative control and a non-treated control was also run.

Plants were allowed to dry at RT until constant weight before preparing extracts as described in^34^. The resulting extract was stored at -20 °C.The resulting extract was stored at -20 °C.

### Phytochemical characterization of RE

#### Total phenolic content (TPC)

Total phenolic content was determined by spectrophotometry using Biomate-5 UB-visible spectrophotometer (Thermo Fisher Scientific). Fifty µL of previously prepared ethanolic extract was used for each analysis. Folin-Ciocalteu method adjusted form Benvenuti et al. (2004) was followed. Folin-Ciocalteu reagent (Sigma-Adlrixh) was added to the extract followed by sodium carbonate (Na_2_CO_3_). This mixture was incubated for 30 min at 25 °C in dark conditions, and the absorbance was then measured at 760 nm. Results of total phenolic content was expressed as milligrams of gallic acid equivalents per gram of dry extract (mg GAE/g) based on standard calibration curve with gallic acid (*r* = 0.992).

### Total flavonol content (TFC)

Total flavonol content was determined following colorimetric method proposed by^50^ with modifications. 1mL of the ethanolic extract of each sample was reacted with aluminum chloride (AlCl_3_) and sodium acetate. Absorbance was measured at 510 nm and quantification was based on a calibration curve prepared with quercetin. Results were expressed as milligrams of quercetin equivalents per gram of dry extract (mg QE/g) (*r* = 0.999).

### HPLC analysis

The chemical composition of the ethanolic extracts was analyzed using a modified version of the protocol described by^51^. The analysis was performed on an Agilent 1100 Series HPLC system equipped with a G1315A diode array detector (DAD). Chromatographic separation was achieved using an Inertsil ODS-3 V column (4.6 × 150 mm, 5 μm particle size, 100 Å pore size; GL Sciences). The mobile phase comprised of:

- **Phase A**: 840 mL Milli-Q water + 8.5 mL acetic acid + 150 mL acetonitrile.
- **Phase B**: 100% methanol.

A linear gradient was applied, decreasing phase A from 90% to 0% over 30 min and the flow rate was set at 1.5 mL/min. Column temperature was maintained at 40 °C. Detection was performed at 284 nm, with a ± 30 nm bandwidth, while full UV absorption spectra were recorded from 200 to 800 nm for each peak to support compound identification. The secondary metabolites carnosic acid and carnosol (diterpenes) and rosmarinic acid (phenolic), were identified based on retention times and spectral profiles, compared to authentic Sigma-Adlrich standards: Carnosic acid (Cat. No. PHR2208-20MG) Carnosol (Cat. No. C9617-5MG) and Rosmarinic acid (Cat. No. 536954-5G), and quantified using calibration curves constructed from standard compounds. Calibration curves for each quantified molecule are shown in Figure SM.5.

### Total antioxidant capacity (TAC)

Total antioxidant capacity (TAC) of *Salvia rosmarinus* Spenn. extracts was measured using the BRS (BQC Redox System, BQC Redox Technologies, Oviedo, Asturias, Spain), following the manufacturer’s instructions^52^.

Every extract was measured three times to obtain reproducible results.

### Data processing and statistical analysis

Extractions and all quantifications were conducted in triplicate to ensure reproducibility and statistical reliability. Values were expressed as mean ± standard deviation (SD) from three independent measurements per treatment. One-way ANOVA was done to evaluate the differences between groups. When the analysis indicated statistical significance (*p* < 0.05), Tukey’s Honestly Significant Difference (HSD) post hoc test was applied to identify pairwise differences. Statistical analyses were conducted using the JASP software (version 0.19.3) [JASP Team, University of Amsterdam].

## Supplementary Information

Below is the link to the electronic supplementary material.

Supplementary Material 1

## Data Availability

The raw data supporting the findings of this study, including nanoparticle synthesis and characterization and phytochemical characterization of postharvest rosemary extracts treated with *Bacillus* G36–derived silver nanoparticles, are publicly available in Zenodo at: DOI 10.5281/zenodo.17977099.
