# Supplementary material for: Postharvest delivery of Bacillus G36 metabolites formulated in AgNP modifies Salvia rosmarinus Spenn. bioactive profiles
Source: Sci Rep. 2026 Mar 17;16:13854. doi: 10.1038/s41598-026-43957-z (PMC13128909; doi:10.1038/s41598-026-43957-z)
Supplement: Supplementary file 1 — Supplementary Material 1 [file 41598_2026_43957_MOESM1_ESM.docx]

Supplementary Materials

Experiment 1


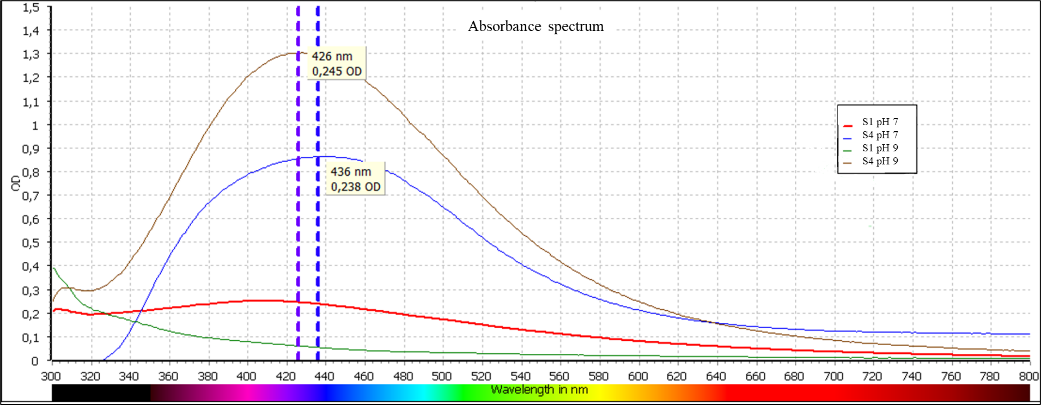


**Figure SM.1.** Characteristics of the biosynthesized AgNPs at pH 7 ratio S1 (red); pH 7 ratio S4 (dark blue); pH 9 ratio S1 (dark green) and pH 9 ratio S4 (brown) with UV-vis spectra.

**
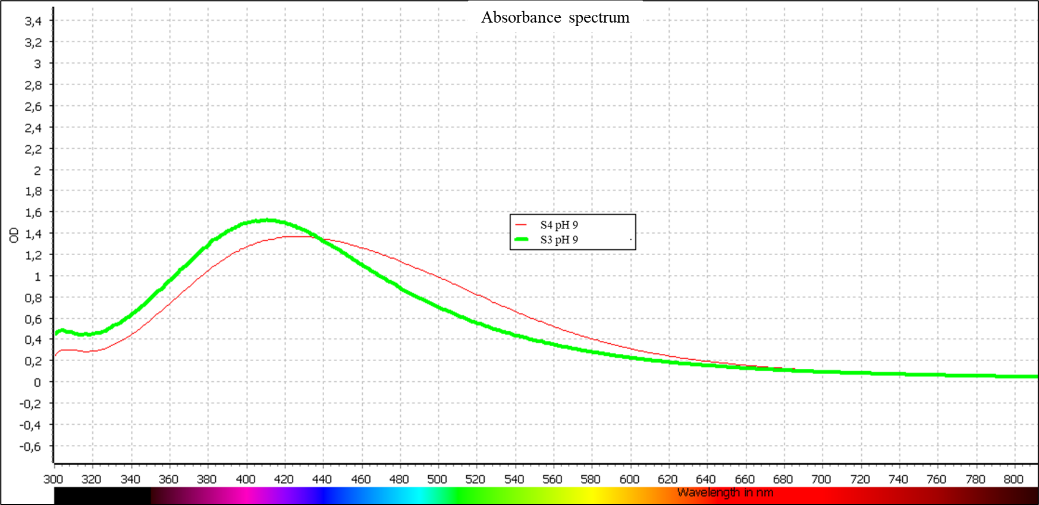
**

**Figure SM.2.** Characteristics of the biosynthesized AgNPs at pH 9 ratio S3 (green) and pH 9 ratio S4 (red) with UV-vis spectra.

Experiment 2


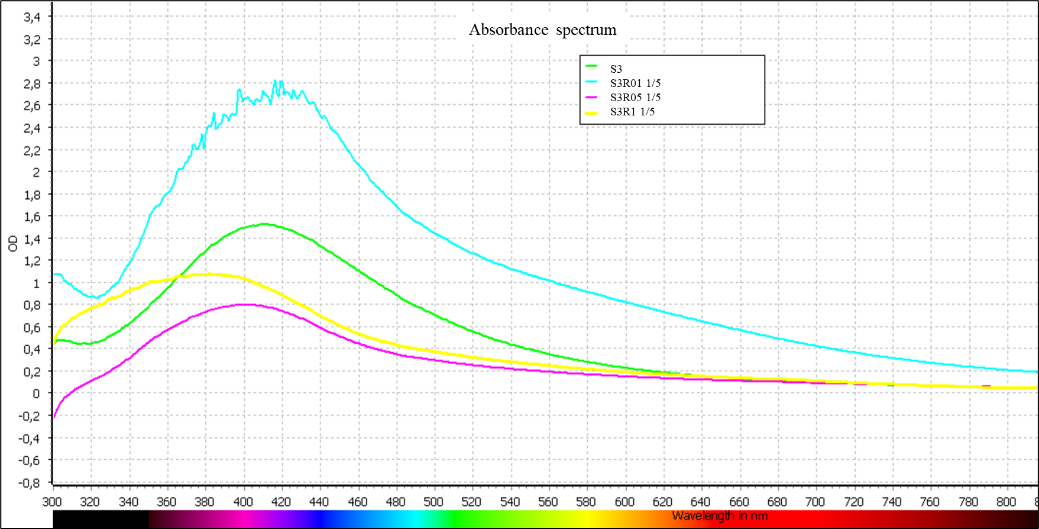


**Figure SM. 3.** Characteristics of the biosynthesized AgNPs: pH 9 ratio S3 (green); pH 9 ratio S3R01 diluted 1/5 (blue); pH 9 ratio S3R05 diluted 1/5 (pink); pH 9 ratio S3R1 diluted 1/5 (yellow) with UV-vis spectra.

Biological assay


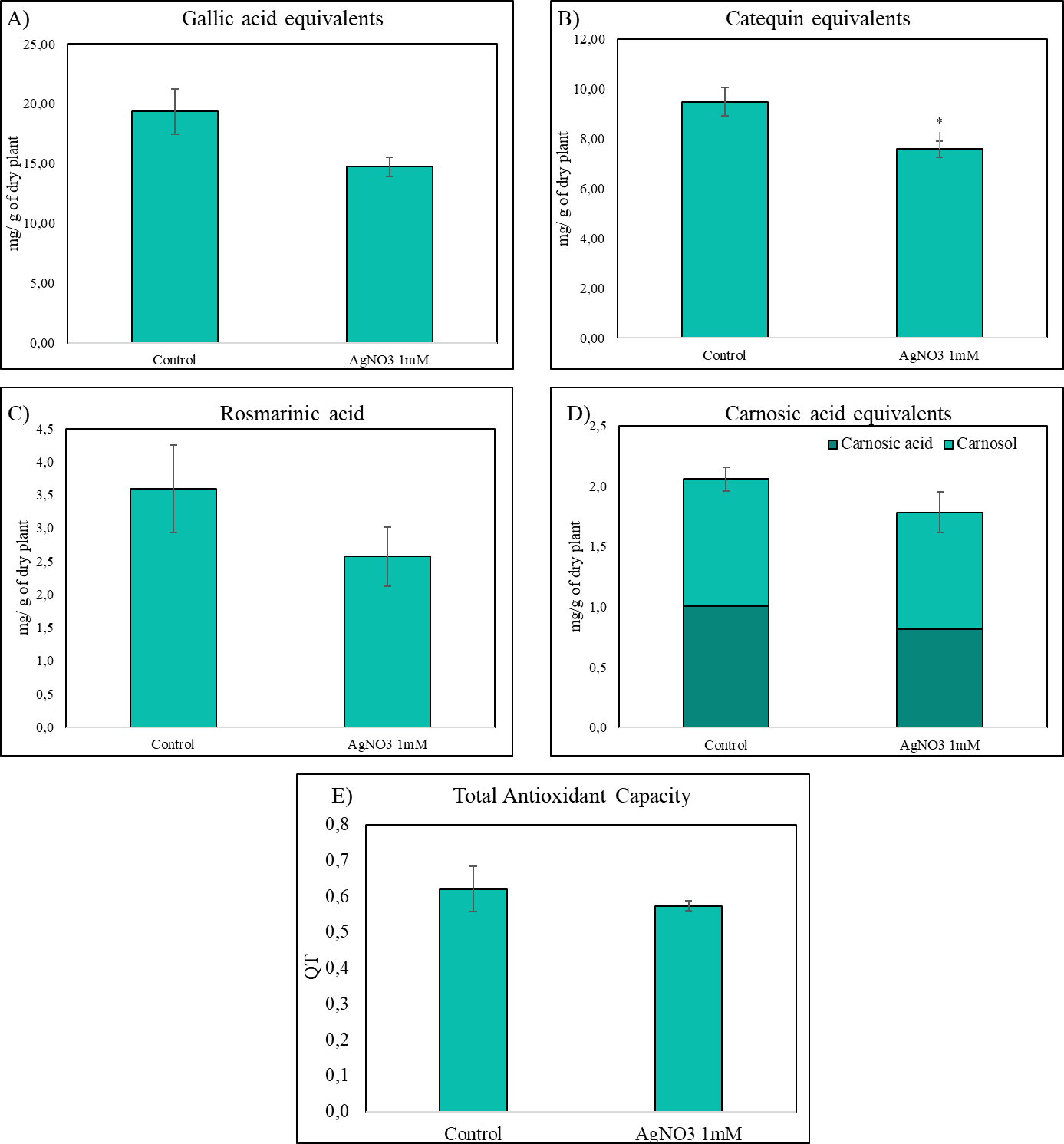


**Figure SM. 4.** A) Gallic acid equivalents and B) catequin equivalents are used to represent A) total phenol and B) flavanols values for rosmarinus extracts under the described treatments. C) Rosmarinic acid quantification expressed as mg of rosmarinic acid per gram of dry plant for different treatments. D) Shown values represent carnosic acid equivalents, determined by summarizing carnosol and carnosic acid concentrations, expressed in mg per gram of dry plant. E) TAC was expressed as QT (total electric charge transferred during the oxidation reaction) in µC. Values correspond to the mean ± SD (n=3), (*) indicateT-student test (p < 0.05).

Calibration curves for HPLC analysis


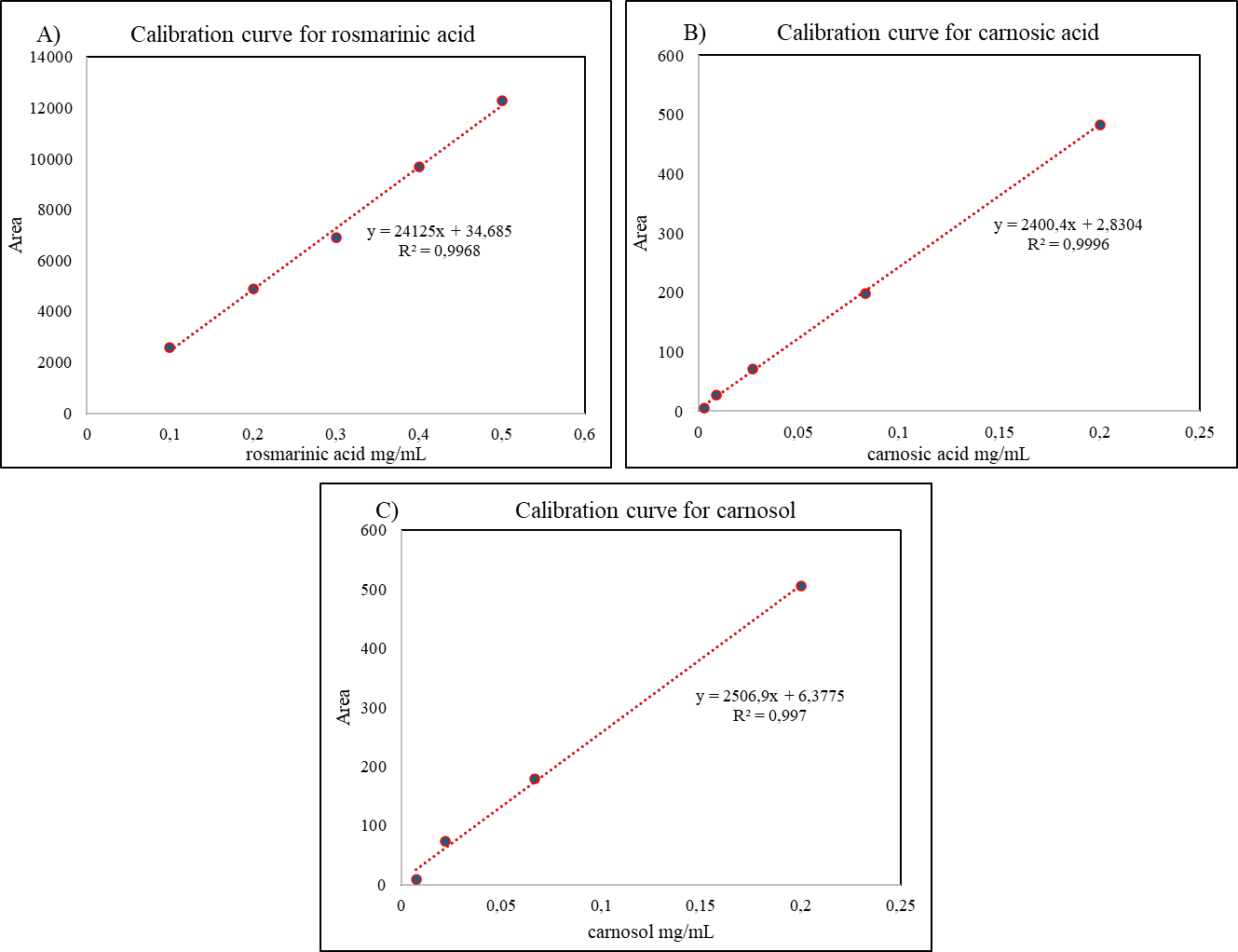


**Figure SM. 5.** Calibration curves for A) rosmarinic acid B) canoscic acid and C) carnosol in HPLC obtained areas respect to different pure standard concentrations (mg/mL).
